# Plasma Fibroblast Growth Factor 23 Is Elevated in Pediatric Primary Hypertension

**DOI:** 10.3389/fped.2019.00135

**Published:** 2019-04-16

**Authors:** Yao Lin, Lin Shi, Yanyan Liu, Hongwei Zhang, Yang Liu, Xiaolan Huang, Dongqing Hou, Mingming Zhang

**Affiliations:** ^1^Department of Pediatric Cardiology, Children's Hospital, Capital Institute of Pediatrics, Beijing, China; ^2^Central Diagnostic Laboratory, Children's Hospital, Capital Institute of Pediatrics, Beijing, China; ^3^Department of Epidemiology, Children's Hospital, Capital Institute of Pediatrics, Beijing, China

**Keywords:** FGF 23, pediatric primary hypertension, blood pressure, left ventricular hypertrophy, cardiovascular disease

## Abstract

Fibroblast growth factor 23 (FGF 23), an endocrine hormone regulating the homeostasis of phosphate and vitamin D, has been shown to play a role in cardiovascular disease. Increased blood FGF 23 is found to be associated with elevated blood pressure in adults. However, measurement of FGF 23 in hypertensive children has not been documented. In this study, a total of 98 children with primary hypertension and 37 controls were recruited, and blood FGF 23 was comparatively investigated. Additionally, FGF 23 levels were compared between the subgroups of patients after hypertensive children were sub-grouped according to their cardiac geometry, hypertension stages, insulin levels, and weight. The case group had a FGF 23 level of 48.99 (16.42), expressed as the median (the interquartile range), significantly higher than the 41.72 (7.05) from the control group (*p* = 0.0002). While no remarkable differences were observed in FGF 23 levels between non-obese and obese hypertensive children, between patients with stage 1 and stage 2 hypertension, or between patients with normal and high insulin levels; hypertensive children with abnormal cardiac geometry had significantly higher levels of FGF 23 than patients with normal cardiac geometry (*p* = 0.0085). Our data revealed for the first time that hypertensive children have higher levels of FGF 23. Further studies are needed to examine if lowering FGF 23 improves the cardiac geometry in hypertensive children with higher FGF 23.

## Introduction

Fibroblast growth factor 23 (FGF 23), primarily produced by osteocytes and osteoblasts in bone, was originally recognized as an endocrine hormone by its canonical function to regulate the homeostasis of phosphate and vitamin D (1); it promotes urinary phosphate excretion, inhibits active vitamin D production and augments active vitamin D catabolism (1–4). Beyond serving as a phosphotropic hormone, FGF 23 is a prognosis predictor of chronic kidney disease (CKD) (5–8). Studies exploring the role of FGF 23 in cardiovascular disease (CVD) have also established FGF 23 as a risk factor, independent of CKD, for CVD (9–16).

Pediatric primary hypertension is on the rise (17, 18). Children with high blood pressure (BP) are not only subject to organ damage such as left ventricular hypertrophy (LVH) (19), but also have a greater probability of developing adult hypertension, a major risk factor for CVD (20, 21). FGF 23 levels have been found to be positively associated with BP in adults (9, 22). However, blood FGF 23 levels in children with primary hypertension have not been documented. This study was designed to comparatively investigate blood FGF 23 levels in children with primary hypertension and those with normal blood pressure.

## Methods

### Ethics Approval and Consent

This study was approved by the Medical Research Review Board of Children's Hospital, Capital Institute of Pediatrics, Beijing, in accordance with the Declaration of Helsinki, the Code of Ethics of the World Medical Association. Written informed parental consent was obtained for all participants.

### Study Subjects and Blood Samples

All subjects, aged 8–17 years, were recruited at our department. Children with primary hypertension formed the case group while those with normal blood pressure were included in the control group. Patients with secondary hypertension or conditions that affect kidneys or the cardiovascular, endocrine, or central nervous systems were excluded. All blood samples were obtained in the morning after overnight fasting. All blood pressure measurements were performed using the auscultation method as recommended by the Fourth Report on the Diagnosis, Evaluation, and Treatment of High Blood Pressure in Children and Adolescents, and the results were used for hypertension diagnosis and stage classification (23). Ambulatory BP monitoring was conducted for the computation of the mean BP during the day and night over a 24 h period using the DMS-ABP System (DM Software), which was used to rule out cases of white coat hypertension and obtain the mean systolic and diastolic pressures. Hypertension was diagnosed when the average systolic and/or diastolic BP was in the ≥95th percentile from the auscultation measurement on at least 3 separate occasions adjusted for gender, age and height. Stage 1 hypertension was diagnosed if a child's BP was greater than the 95th percentile but less than or equal to the 99th percentile plus 5 mm Hg; and stage 2 hypertension was diagnosed if a child's BP was greater than the 99th percentile plus 5 mm Hg (23).

### Echocardiography

LVH was assessed by echocardiography. Left ventricular internal dimension (LVIDd), interventricular septal thickness (IVST) and left ventricular posterior wall thickness (LVPWT) at end-diastole were measured using the Philips iE33 Ultrasound System. Relative left ventricular wall thickness (RWT) was calculated as RWT = (IVST+LVPWT)/LVIDd; left ventricular mass (LVM) was calculated as LVM = 1.04 × 0.8 × ((LVIDd+IVST+LVPWT)^3^–LVIDd^3^)+0.6 (24); and LVM index (LVMI) was calculated as LVMI = LVM/height^2.7^. We adopted the criteria proposed by Hietalampi et al. for the diagnosis of abnormal cardiac geometry (25), namely, LVMI ≥ 37.08 g/m^2.7^ in males, LVMI ≥ 34.02 g/m^2.7^ in females or RWT > 0.36 in either males or females is considered abnormal.

### Plasma FGF 23 Measurement

Plasma FGF 23 levels were determined by an enzyme-linked immunosorbent assay using the Human FGF 23 (C-Term) ELISA Kit that measures the C-terminal fragment of FGF 23 (cFGF 23). The assay was performed and the FGF 23 level was expressed as the relative unit/ml (RU/ml) according to the manufacturer's instructions (Quidel Corporation, San Diego, USA).

### Fasting Insulin Measurement

Fasting insulin was measured in the diagnostic laboratory of our hospital following a routine protocol.

### Statistical Analysis

Data normality was determined by the Shapiro-Wilk test. Parametric continuous data were expressed as mean ± standard deviation (SD) and analyzed by two-tailed Student's *t*-test. Non-parametric data were presented as median (the interquartile range) and analyzed by the Mann-Whitney test. *p* < 0.05 was considered statistically significant. All statistical analyses were performed using the SPSS 17.0 software (IBM Corporation, Armonk, USA).

## Results

### Demographics and the Blood Pressure of all Subjects

A total of 98 hypertensive and 37 normotensive children, aged 8 to 17 years, were recruited. Demographics and blood pressures of all subjects are shown in Table 1. There is no substantial difference in average ages between the 2 groups. A previous report revealed that adolescents aged 12–15 years had higher levels of FGF 23 (26). In our series, the proportion of adolescents aged 12–15 years in the control group was 51.4% (19/37), similar to that in the case group (52.0%, 51/98). The case group had a significantly higher body mass index (BMI) than the control group. Both mean systolic and diastolic pressures in the case group were significantly higher than those in the control group (Table 1).

**Table 1:** Demographics and blood pressures of all subjects.

	**Case group** **(*n* = 98)**	**Control group** **(*n* = 37)**
Age (years)	11.8 ± 2.2	11.1 ± 2.2
Number of males (%)	78 (79.59)	11 (29.73)
BMI (kg/m^2^)	27.32 ± 5.36^*^	18.01 ± 2.48
Mean systolic pressure (mmHg)	128.33 ± 12.39^*^	117.73 ± 7.03
Mean diastolic pressure (mmHg)	72.69 ± 12.36^**^	68.35 ± 5.58

*Mean systolic pressure and mean diastolic pressure were obtained from the 24-hour ambulatory blood pressure measurement. Data are expressed as mean ± standard deviation*;

* p = 0.0001 and

** *p = 0.0024 compared with control group*.

*BMI, body mass index*.

### Blood FGF 23 Levels Between Normotensive and Hypertensive Children

All FGF 23 levels were presented in the unit of RU/ml. The FGF 23 level in the case group was significantly higher than that of the control group (*p* = 0.0002, Table 2).

**Table 2:** Comparison of FGF 23 levels (RU/ml) between the case and control groups.

	**Case group (*n* = 98)**	**Control group (*n* = 37)**	***p*-value**
FGF levels	48.99 (16.42)	41.72 (7.05)	0.0002

*FGF levels were presented as median (the interquartile range). P value was determined by the Mann-Whitney test*.

### Blood FGF 23 Levels Between Subgroups of Patients

FGF 23 levels were compared between subgroups of patients. As shown in Table 3, there were no significant differences in FGF 23 levels between non-obese and obese hypertensive children, between patients with stage 1 and stage 2 hypertension, or between patients with normal and high insulin levels (Table 3). However, hypertensive children with abnormal cardiac geometry had significantly higher levels of FGF 23 than patients with normal cardiac geometry (Table 3).

**Table 3:** Comparison of FGF 23 levels (RU/ml) between subgroups of patients.

**Subgroup** **FGF 23 levels**	**Subgroup** **FGF 23 levels**	***p*-value**
Non-obese (*n* = 35) 49.05 (19.08)	Obese (*n* = 63) 48.92 (14.75)	0.52
Stage 1 (*n* = 68) 48.52 (17.44)	Stage 2 (*n* = 30) 51.36 (19.68)	0.28
Normal insulin (*n* = 56) 48.36 (17.68)	High insulin (*n* = 17) 53.66 (22.97)	0.44
Normal cardiac geometry (*n* = 63) 48.08 (17.48)	Abnormal cardiac geometry (*n* = 35) 55.01 (20.18)	0.0085

*Data are presented as median (the interquartile range). P value was determined by the Mann-Whitney test*.

## Discussion

In the present study, we comparatively investigated blood FGF 23 levels in hypertensive and normotensive children, and reported that (1) children with primary hypertension had significantly higher levels of FGF 23 than controls; (2) FGF 23 levels in patients with abnormal cardiac geometry were markedly higher than those in patients with normal cardiac geometry; and (3) there were no significant differences in FGF 23 levels between non-obese and obese hypertensive children, between patients with stage 1 and stage 2 hypertension, or between patients with normal and high insulin levels. Previous studies have shown that FGF 23 levels does not differ between genders (26–28), which was also observed in our study (data not shown).

FGF 23 is a protein consisting of 251 amino acids with an N-terminal region that contains the conserved FGF homology domain and a C-terminus of 71-amino acid (3). There are 2 forms of FGF 23 identified in circulation: one is the intact FGF 23 and the other is the C-terminal fragment of FGF-23 that is created by proteolysis (29). It has been shown that cFGF-23 is biologically active, e.g., it retains phosphaturic activity (29). The cFGF23 ELISA kit detects both intact FGF 23 and cFGF 23 and is preferentially used in the measurement of blood FGF 23 (26). In view of these facts, we chose the cFGF23 ELISA kit for this study. FGF23 blood levels are independent of gender in the pediatric population (26–28). Therefore, the cases and controls were matched only by age in this study.

As one of the most important regulators of blood phosphate, FGF 23 stimulates urinary phosphate excretion. It has been shown in both adult and pediatric patients that FGF 23 levels rise as kidney function declines (5–8, 25, 27, 28, 30). As a marker of CVD, circulating FGF 23 is found positively related to blood pressure in adults (9, 22). In a case-control study, Gutiérrez et al. found that adults with hypertension had significantly higher FGF23 than those without hypertension (9). In a prospective observational study exploring the associations of FGF 23 levels with longitudinal BP changes and incident hypertension in young and middle-aged adults, Akhabue et al. revealed that at the 5 and 10-year-follow-up visits the subjects in the highest FGF 23 quartile had a greater chance to develop hypertension than those in the lowest FGF 23 quartile, indicating that higher FGF 23 levels increase the risk of incident hypertension (22). In the present study, we observed increased plasma FGF 23 levels in pediatric patients with primary hypertension. To date, circulating FGF 23 measurement in hypertensive children has not been documented. Zaja̧c et al. discovered that hypertensive children and adolescents had significantly higher urine FGF 23 levels than controls, but they did not measure blood FGF 23 (31). We speculate that higher urine FGF 23 concentrations in hypertensive participants might be a result of increased blood FGF 23 levels.

FGF 23 has been shown to be independently associated with left ventricular mass, hypertrophy and geometry in adult patients with or without CKD (13–15, 32). Additionally, in animal studies direct hypertrophic effects of FGF 23 on isolated neonatal rat ventricular cardiomyocytes is observed (13). It has also been demonstrated that direct injection of FGF 23 protein into the left ventricular myocardium of mice results in LVH (13). These findings support a causal role of FGF 23 in LVH. In contrast, Agarwal et al. revealed that FGF 23 was not associated with LVH in patients with coronary artery disease (33). We reported here that children with abnormal cardiac geometry had remarkably higher blood FGF 23 levels than those with normal cardiac geometry. This finding is in agreement with the data presented in a study showing that FGF 23 values were significantly higher in adolescents with eccentric or concentric LVH compared with those without hypertrophy (34). The increase of FGF 23 found in our study is unlikely a result of kidney disease as all participants had normal kidney function. Further studies to examine if lowering FGF 23 improves cardiac geometry in those with higher FGF 23 will help uncover if FGF 23 plays a determinant role in LVH in children.

Hypertensive children have a higher rate of obesity (23, 35, 36), which is also seen in this study. Elevated FGF 23 levels have been documented in obese vs. non-obese adults and adolescents as well (34, 37). Ali et al. found that obese adolescents or adolescents with higher fasting insulin concentrations had elevated FGF 23 in a study population consisting exclusively of African Americans (34). In contrast, our results showed that there were no significant differences in FGF 23 levels between obese and non-obese hypertensive children, or between patients with normal and high insulin levels. These discrepancies might be due to racial differences.

Considering that this is a small-scale study from a single center, the generalizability of our data might be limited. Additionally, the control group is about a third the size of the cases, which might limit the statistical power. Further studies in a large series and in other race and ethnic groups are needed.

## Author Contributions

All authors listed have made a substantial, direct and intellectual contribution to the work, and approved it for publication.

### Conflict of Interest Statement

The authors declare that the research was conducted in the absence of any commercial or financial relationships that could be construed as a potential conflict of interest.
